# Evaluating AI-Mediated Health Communication via Large Language Model–Based Frequently Asked Questions Rewriting to Foster Clinical Trial Participation: Comparative Survey Study

**DOI:** 10.2196/87446

**Published:** 2026-05-12

**Authors:** Ching-Hua Chuan, Jiajing Tang, Zixiao Yang, Rutendo Chimbaru

**Affiliations:** 1 Department of Interactive Media, School of Communication, University of Miami, 5100 Brunson Drive, Coral Gables, FL, 33146, United States, 1 3052845045; 2 Department of Communication, School of Humanities & Social Sciences, Emporia State University, Emporia, KS, United States

**Keywords:** large language models, clinical trial participation, Theory of Planned Behavior, frequently asked questions, artificial intelligence–mediated health communication, AI-mediated health communication

## Abstract

**Background:**

Effective communication about clinical trials is essential, as low enrollment undermines scientific validity and contributes to health care inequities. However, recruitment remains a persistent challenge, particularly among older adults, minority populations, and individuals with limited health literacy. Although large language models (LLMs) show promise in understanding and generating health information, it is unclear whether these generative artificial intelligence (AI) tools can improve the content of hospitals’ frequently asked questions (FAQ) pages to enhance public attitudes and intentions toward clinical trial participation.

**Objective:**

This study aimed to compare clinical trial FAQ from health organizations and hospitals with versions rewritten by LLMs to examine whether the generated content improves public attitudes and intentions toward clinical trial participation and to identify the mechanisms underlying these effects.

**Methods:**

A total of 308 question-answer pairs were collected from the FAQ pages of 38 health organizations and hospitals, categorizing them into 52 types and selecting the 11 most frequent for testing. A comparative survey experiment was conducted with 440 participants randomly assigned to one of the two survey stimuli: the original FAQ versus the GPT-4o–generated answers emphasizing comprehension and empathy. The study compared the impact of AI-generated versus standard FAQ content on attitudes toward clinical trials and examined Theory of Planned Behavior constructs to determine for whom and how AI information is most effective.

**Results:**

Participants were recruited through CloudResearch, yielding a 96.94% completion rate, resulting in 440 valid responses across the 2 types of content exposure. Participants who viewed GPT-4o–generated information (mean 0.26, SD 0.65) showed a marginally greater positive change in outcome evaluation attitudes than those who viewed standard FAQ (mean 0.13, SD 0.70; *P*=.05; 95% CI 0.00-0.25). Follow-up linear regression analyses revealed that several individual factors significantly moderated the effect of the information type (FAQ vs GPT-4o) on attitude change, including age (mean difference 0.87, SE 0.33; 2-tailed *t*_394_=2.62; *P*=.009); race (mean difference 0.36, SE 0.15; *t*_383_=2.47; *P*=.01); risk aversion (B=0.12; SE 0.06; *t*_383_=2.23; *P*=.03); fear of ineffective treatment (B=0.11; SE 0.05; *t*_383_=2.03; *P*=.04); and fear of unknown treatment effects (B=0.21; SE 0.07; *t*_383_=3.10; *P*=.002).

**Conclusions:**

This study is the first to apply the Theory of Planned Behavior to compare LLM-rewritten versus original FAQ content for clinical trial communication. The findings show that the GPT-4o–generated responses improved attitudes among traditionally underrepresented groups, including older adults, Black participants, and those with higher uncertainty avoidance or treatment concerns. These attitude gains were positively linked to participation intentions, suggesting that AI-generated language can enhance public attitudes, perceptions, and engagement with clinical research.

## Introduction

Clinical trials are essential for evaluating the safety and effectiveness of new drugs and treatments across diseases, including cancer. However, persistently low enrollment rates among certain populations limit the generalizability of research findings [1-4]. Prior studies have identified multiple barriers to participation, with many stemming from how information is presented and perceived. For instance, the study by Clark et al [5] reported that mistrust of investigators and insufficient information hinder participation by minority groups. The study by Ridda et al [6] found that lengthy or complex explanations increase older adults’ concerns about participation. Similarly, the study by Simon et al [7] highlighted that limited health and research literacy, that is, unfamiliarity with clinical trial purposes and procedures, reduces individuals’ willingness to enroll.

Recent advances in artificial intelligence (AI), particularly large language models (LLMs) such as GPT-4o, present new opportunities to enhance health communication. LLMs can bridge language gaps, promote transparency through translation [8], and simplify complex medical information and clinical documentation [9,10]. A rather underexplored benefit of LLMs in health care is their potential to adapt messages to individuals’ communication styles [11,12]. However, it remains unclear whether and how LLM-generated messages can increase public awareness of health issues, influence health behaviors, or enhance intentions to participate in health-related activities such as clinical trials. Although some studies have explored this topic [13], most have focused on message perception rather than on the mechanisms underlying LLM effectiveness.

To address this research gap, this study draws on the Theory of Planned Behavior (TPB), which posits that individuals’ attitudes, perceived social norms, and perceived behavioral control shape their intentions to engage in specific behaviors [14]. Attitudes reflect overall evaluations of a behavior (eg, viewing clinical trial participation as beneficial or risky); perceived norms capture the influence of others’ opinions, such as encouragement from family, peers, or health care providers. Perceived behavioral control reflects confidence in one’s ability to perform the behavior, including understanding and managing clinical trial information. In the context of LLM-mediated communication, the TPB provides a useful framework not only to assess changes in attitudes and intentions but also to identify for whom AI-generated information is most effective. Background factors such as age, race, and health literacy may influence these effects. Accordingly, this study examined how TPB constructs interact to determine for whom and in what ways AI-generated responses shape behavioral intentions toward clinical trial participation.

## Methods

### Stimuli Design

To compile clinical trial information available to the public, 308 question-answer pairs were collected from the frequently asked questions (FAQ) pages of 38 health organizations and hospitals. One author reviewed and categorized the questions into 52 types following the framework outlined in the study by Peng et al [15], encompassing topics such as trial procedures, safety, financial considerations, and eligibility criteria. Questions were then ranked by frequency, and the 11 most common questions were selected for the experiment. The questions, their sources, and corresponding GPT-4o–generated answers are provided in Multimedia Appendix 1.

A comparative survey experiment was conducted with participants recruited online. Participants were given one of the 2 stimuli randomly: participants either viewed selected questions and their corresponding answers from existing FAQ pages or viewed the same questions accompanied by answers generated by GPT-4o. The answers were generated by prompting the model to produce responses emphasizing ease of comprehension, empathy, and a patient-centered tone, with the setting of the temperature parameter to 0.2. The prompt used was as follows:

You are a medical professional. Answer the question below about clinical trials in an easy-to-understand way with empathy. The language needs to be brief (within 50 words) and clear. The goal is to encourage people’s participation by providing positive evidence, addressing individuals’ concerns, and emphasizing the actions people can take in the process. Rewrite the question to the sixth-grade comprehension level if it is not. Question: “What happens in a clinical trial?”

The GPT-4o–generated answers were manually reviewed for accuracy by an author experienced in clinical trial recruitment research. To validate the manipulation, the generated and original FAQ answers were compared using established readability and tone metrics. Readability was assessed using the Simple Measure of Gobbledygook Index [16], the Flesch Reading Ease formula [17], and the Flesch Reading Ease score [18], all of which estimate the grade level or difficulty required to comprehend the text. Scores were computed with the textstat Python package [19]. Tone valence was evaluated using the sentiment intensity analyzer in the Natural Language Toolkit [20], which produces values from −1 (negative) to +1 (positive). As shown in Table 1, GPT-4o–generated answers demonstrated lower required reading levels, greater reading ease, and more positive tones than the original FAQs. Notably, the average Flesch-Kincaid grade level for the FAQ content (grade level 11) was approximately one grade lower than that reported for informed consent forms from nearly 800 federally funded trials (grade 12) [21].

**Table 1.** Readability and sentiment scores on frequently asked questions (FAQ) and the GPT-4o–rewritten content.

Model	Simple Measure of Gobbledygook index	Flesch-Kincaid grade level	Flesch Reading Ease score	Sentiment score
FAQ	12	11	49.54 (difficult)	0.39
GPT-4o	10	7	62.72 (standard)	0.88

### Recruitment

A between-subject survey experiment was conducted with 440 participants recruited through the CloudResearch Connect platform between May 6 and May 12, 2025. Eligibility criteria required that participants be US residents aged ≥18 years, demonstrate high English proficiency, and pass attention check questions. The online survey took approximately 20 minutes to complete.

### Ethical Considerations

The study protocol was approved by the institutional review board of the University of Miami (20231288). Participants received US $4 for completing the study. Prior to completing the survey, participants were provided with an informed consent form outlining the study’s purpose, potential risks and benefits, and compensation. They were also informed that participation was voluntary and that they could withdraw from the study at any time. No identifiable information was collected in the study.

### Measurements

#### Core Constructs in the TPB

Preattitudes and postattitudes toward participating in clinical trials were assessed using 2 dimensions: behavioral beliefs (for pre: mean 5.17, SD 1.02; for post: mean 5.34, SD 1.06) and evaluation outcomes (for pre: mean 5.48, SD 1.26; for post: mean 5.67, SD 1.28). These scales were adapted from prior research in health settings [22]. Behavioral beliefs were measured using a 7-point Likert scale (1=*very unlikely*; 7=*very likely*). The following is a sample item:

My participation in a clinical trial will improve my health.

Evaluation outcomes were assessed using a 7-point Likert scale following the statement, “I believe participating in a clinical trial is...,” with bipolar adjective pairs such as good-bad and beneficial-harmful. These items were reversed coded such that higher scores indicate favorable evaluation of clinical trial participation.

Preinjunctive and postinjunctive norm (for pre: mean 3.99, SD 1.32; for post: mean 4.11, SD 1.32) was measured using 4 items on a 7-point Likert scale (1=*strongly disagree*; 7=*strongly agree*) [22]. The following is a sample item:

The people in my life whose opinions I value would approve of me participating in a clinical trial.

Other items included the following:

It is expected of me to participate in clinical trials when invited.

Members of my community would think that I should participate in clinical trials.

My friends would think that I should participate in clinical trials.

Predescriptive and postdescriptive norm (for pre: mean 5.05, SD 1.24; for post: mean 5.34, SD 1.29) was measured using 2 items adopted from the study by Ajzen [23]. Participants rated their agreement on a 7-point Likert scale (1=*strongly disagree*; 7=*strongly agree*). The items assessed perceptions of how commonly others engage in clinical trial participation. The following are sample items:

Many people participate in clinical trials when they need treatment.

People seeking treatment tend to participate in clinical trials.

Preperceived and postperceived behavioral control (for pre: mean 5.31, SD 1.21; for post: mean 5.68, SD 1.14) was assessed using 4 items [22]. Participants indicated their agreement on a 7-point Likert scale (1=*definitely fals*e; 7=*definitely true*). The items measured participants’ perceived ease, confidence, and autonomy in deciding whether to participate in a clinical trial. The following is a sample item:

It will be very easy for me to participate in a clinical trial if I wanted to.

#### Individual Background Factors

Background variables were measured to understand how individuals’ background affects their attitudes and perceptions toward clinical trials, and they were collected at the end of the experiment to avoid priming effects.

Perceived fear toward clinical trial participation was measured using separate items, with each representing a specific concern as an important barrier to their participation in a clinical trial [24]. Participants rated each item on a 5-point Likert scale (1=*least important factor*; 5=*most important factor*). The following is a sample item:

That the experimental treatment can cause damage to my health.

An open-ended option (“Other, please specify”) was also provided but not required. The mean scores for these types of fear range from 2.07 to 4.17, with SDs ranging from 1.00 to 1.42.

Health literacy (mean 4.34, SD 0.88) was measured using 4 items adapted from previously validated health literacy assessments [25]. Participants rated each item on a 5-point Likert scale (1=*none of the time*; 5=*all of the time*). The items assessed individuals’ ability to understand and manage health-related information. The following is a sample item:

How often do you have someone help you read hospital materials?

Some items were reverse coded so that higher scores indicate greater perceived health literacy.

Participants’ prior health care experience and perception were measured through trust in the health care system, their perceived discrimination in health care, and their prior experience with clinical trials.

Trust in the health care system (mean 3.59, SD 0.89) was measured using 4 items adapted from previous studies [26,27]. Participants rated their agreement with each statement on a 5-point Likert scale (1=*not at all*; 5=*very*). The items assessed participants’ confidence and perceived reliability of the health care system. The following are sample items:

How much do you trust the health care system?

How confident are you in the health care system’s ability to care for your health?

Higher scores indicate greater trust in the health care system.

Perceived discrimination in health care (mean 2.90*,* SD 1.08) was measured using 4 items adapted from the study by Hausmann et al [28]. Participants rated their agreement with each statement on a 5-point Likert scale (1=*strongly disagree*; 5=*strongly agree*). The following are sample items:

Doctors treat African American and White people the same.

In most hospitals, African American and Whites receive the same kind of care.

Racial discrimination in a doctor’s office is common.

Some items were reverse coded such that higher scores indicate higher perceived discrimination.

Prior experience with clinical trials was measured through a multiple-select question (select all that apply), indicating participants’ prior experience with clinical trials from 4 items:

I participated in a clinical trial before or I am currently enrolled in a clinical trial.

I know someone (a family member or a friend) participated in a clinical trial.

I have family members who were diagnosed with cancers.

Neither me nor people I know have participated in a clinical trial.

Participants’ cultural background was measured through Hofstede dimensions [29], which were adopted for medical psychology and health care in the study by Meeuwesen et al [30]. In this study, Hofstede culture dimensions were adopted in 3 measures: uncertainty avoidance, collectivism, and power distance:

Uncertainty avoidance (mean 5.26, SD 1.19) was measured using 5 items on a 7-point Likert scale (1=*strongly disagree*; 7=*strongly agree*) adapted from the study by Astvansh et al [31]. The scale assessed participants’ discomfort with ambiguity and preference for predictability in decision-making contexts. The following are sample items:

I prefer structured situations to unstructured situations.

I tend to get anxious easily when I don’t know the outcome.

Collectivism (mean 3.97*,* SD 1.45) was measured using 6 items on a 7-point Likert scale (1=*strongly disagree*; 7=*strongly agree*) adapted from the study by Yoo et al [32]. The items assessed participants’ perception on the importance of groups or communities over individual interests. The following are sample items:

Individuals should sacrifice self-interest for the community they are in.

The welfare of the community where individuals belong to is more important than individual rewards.

Higher scores indicate greater values on the group or communities over individual interests.

Patient power distance (mean 2.54*,* SD 0.70) was measured using 7 items on a 5-point Likert scale (1=*strongly disagree*; 5=*strongly agree*), adapted from the perception of authority subscale in the study by Wang et al [33]. The following are sample items:

I don’t think patients need to question medical decisions made by medical staff.

I was nervous when I told the medical staff what was really bothering me.

Higher scores indicate greater power distance perceived by the participant from a patient’s perspective.

Demographics were asked of participants to report their gender, age, race, ethnicity, and primary language spoken at home. Additional variables included educational levels, marital status, employment status, and annual household income.

### Statistical Analysis

The data analysis was conducted using R (version 4.2.2; R Foundation for Statistical Computing). Reliability of the multi-item scales measuring pre- and postattitude (behavioral belief), attitude (outcome evaluation), injunctive norm, descriptive norm, perceived behavioral control, and individual background factors (eg, health literacy, trust in the health care system, and uncertainty avoidance) was examined and found to be acceptable, with Cronbach *α* values ranging from 0.71 to 0.94. Average scores were used to compute these variables.

Change scores for attitude (behavioral belief), attitude (outcome evaluation), injunctive norm, descriptive norm, and perceived behavioral control were calculated by subtracting the prescore from the postscore. A binary variable representing positive intention change was created, coded as 1=positive change and 0=else. Specifically, 1 was assigned to participants who answered “no” or “don’t know” to the participation intention question before the content exposure and “yes” after reading the content.

Descriptive statistics were reported using frequency and percentage for categorical variables, and mean and SD for continuous variables. Descriptive statistics were first examined for prescores, postscores, and change scores of attitudes, norms, perceived behavioral control, and intention across the 2 stimuli or information models (GPT-4o vs FAQ). To test whether differences were statistically significant, a multivariate ANOVA was conducted for the TPB-related variables, including attitudes, norms, and perceived behavioral control. A series of linear regression models were then used to explore whether the effects of the information model on these changes varied depending on individual factors by introducing interaction terms. Binary logistic regression analyses were conducted to examine whether the information model (GPT-4o vs FAQ), along with TPB-related variables, significantly influenced the positive intention change. Finally, pathway analyses were performed to further confirm the initial findings suggested by the regression models. *P* values <.05 were considered statistically significant.

## Results

### Characteristics of the Participants

The final sample consisted of 440 participants, all of whom passed the attention check questions. Most participants were aged between 25 and 54 years (359/440, 82%), and gender distribution was nearly balanced, with 223 (N=440, 51%) men and 212 (N=440, 48%) women. The majority identified as non-Hispanic or non-Latino (407/440, 93%). In terms of race, most participants identified as White (301/440, 68%), followed by African American or Black (85/440, 19%), Asian or Pacific Islander (32/440, 7%), mixed race or multiracial (18/440, 4%), other (3/440, 0.7%), and Native American or American Indian (1/440, 0.2%). Nearly all participants reported English as their primary language (436/440, 99%). Additional participant characteristics, as well as their distributions across the FAQ and GPT-4o stimuli, are presented in Table 2.

**Table 2.** Basic characteristics of participants (N=440).

Variables	Total, n (%)	GPT-4o (n=220), n (%)	FAQ^a^ (n=220), n (%)
Age group (years)			
18‐24	32 (7)	13 (6)	19 (9)
25‐34	143 (33)	66 (30)	77 (35)
35‐44	137 (31)	69 (31)	68 (31)
45‐54	79 (18)	43 (20)	36 (16)
55‐64	28 (6)	15 (7)	13 (6)
>65	21 (5)	14 (6)	7 (3)
Gender			
Man	223 (51)	107 (49)	116 (53)
Woman	212 (48)	111 (50)	101 (46)
Nonbinary	3 (0.7)	2 (0.9)	1 (0.5)
Prefer not to say	2 (0.5)	0 (0)	2 (0.9)
Ethnicity			
Hispanic or Latino	33 (8)	12 (5)	21 (10)
Non-Hispanic or non-Latino	407 (93)	208 (95)	199 (90)
Race			
African American or Black	85 (19)	42 (19)	43 (20)
Asian or Pacific Islander	32 (7)	13 (6)	19 (9)
Caucasian or White	301 (68)	155 (70)	146 (66)
Native American or American Indian	1 (0.2)	1 (0.5)	0 (0)
Mixed race or multiracial	18 (4)	8 (4)	10 (5)
Other	3 (0.7)	1 (0.5)	2 (0.9)
Primary language			
English	436 (99)	219 (99.5)	217 (98.6)
Spanish	0 (0)	0 (0)	0 (0)
Other	2 (0.5)	0 (0)	2 (0.9)
Prefer not to say	2 (0.5)	1 (0.5)	1 (0.5)
Education			
High school or less	44 (10)	20 (9)	24 (11)
Some college (no degree)	75 (17)	36 (16)	39 (18)
Technical certification	10 (2)	5 (2)	5 (2)
Associate degree (2 years)	43 (10)	18 (8)	25 (11)
Bachelor’s degree (4 years)	193 (44)	105 (48)	88 (40)
Master’s degree	63 (14)	30 (14)	33 (15)
Doctoral degree	6 (1)	4 (2)	2 (0.9)
Professional degree (JD and MD)	6 (1)	2 (0.9)	4 (2)
Marital status			
Married	217 (49)	115 (52)	102 (46)
Widowed	8 (2)	5 (2)	3 (1)
Divorced	29 (7)	15 (7)	14 (6)
Separated	4 (0.9)	3 (1)	1 (0.5)
Never married	182 (41)	82 (37)	100 (45)
Employment status			
Full time	281 (64)	141 (64)	140 (64)
Part time	56 (13)	31 (14)	25 (11)
Contract or temporary	15 (3)	9 (4)	6 (3)
Retired	11 (3)	8 (4)	3 (1)
Unemployed	46 (10)	22 (10)	24 (11)
Unable to work	5 (1)	3 (1)	2 (0.9)
Other	23 (5)	6 (3)	17 (8)
Prefer not to say	3 (0.7)	0 (0)	3 (1)
Income (US $)			
0-29,999	66 (15)	33 (15)	33 (15)
30,000-59,999	150 (34)	87 (40)	63 (29)
60,000-89,999	86 (20)	40 (18)	46 (21)
90,000-119,999	47 (11)	23 (10)	24 (11)
>120,000	91 (21)	37 (17)	54 (25)

a FAQ: frequently asked questions.

Chi-square tests were conducted to examine the distribution of participant characteristics across the two information models. The results indicated no significant differences in these factors (including age, gender, ethnicity, race, primary language, education, marital status, employment status, and income) between the two information models (all *P* values >.05), suggesting baseline equivalence.

### Effects on Changes in Attitudes, Norms, and Perceived Behavioral Control

As shown in Table 3, mean scores for attitudes (both behavioral belief and outcome evaluation), norms (both injunctive and descriptive), and perceived behavioral control toward clinical trial participation increased after exposure to the question and answer content compared with before, across both the GPT-4o and FAQ stimuli. However, results from a 1-way multivariate ANOVA indicated that the overall differences in mean change scores across these variables were not significant between the two information models (Pillai trace=0.02; *F*_5,434_=1.66; *P*=.14; partial η²=0.02). Follow-up univariate tests were also nonsignificant for all individual change variables (all *P* values >.05), although the effect of the information model on the change in attitude (outcome evaluation) was marginally significant. Specifically, participants who viewed the GPT-4o–generated information showed a marginally greater positive change in this variable than those who viewed the FAQ version (mean difference 0.12, SE 0.06; *F*_1,438_=3.66; *P*=.05; 95% CI 0.00-0.25).

**Table 3.** Changes in attitudes, norms, and perceived behavioral control (N=440).

Variables	Total, mean (SD)	GPT-4o (n=220), mean (SD)	FAQ^a^ (n=220), mean (SD)	Difference (95% CI)^b^	*F* test (*df=*1,438)^c^	*P* value	η²
Attitude (behavioral belief)				0.08 (−0.07 to 0.23)	1.10	.29	0.003
Pre	5.17 (1.02)	5.17 (0.95)	5.17 (1.08)				
Post	5.34 (1.06)	5.38 (1.04)	5.30 (1.09)				
Change	0.17 (0.79)	0.21 (0.78)	0.13 (0.79)				
Attitude (outcome evaluation)				0.12 (0.00 to 0.25)	3.66	.05	0.008
Pre	5.48 (1.26)	5.44 (1.25)	5.51 (1.26)				
Post	5.67 (1.28)	5.70 (1.28)	5.64 (1.27)				
Change	0.20 (0.68)	0.26 (0.65)	0.13 (0.70)				
Injunctive norm				−0.05 (−0.19 to 0.08)	0.57	.45	0.001
Pre	3.99 (1.32)	4.16 (1.28)	3.83 (1.34)				
Post	4.11 (1.32)	4.25 (1.23)	3.97 (1.40)				
Change	0.12 (0.73)	0.09 (0.79)	0.14 (0.66)				
Descriptive norm				−0.11 (−0.30 to 0.08)	1.22	.27	0.003
Pre	5.05 (1.24)	5.13 (1.15)	4.97 (1.32)				
Post	5.34 (1.29)	5.37 (1.22)	5.31 (1.36)				
Change	0.29 (1.02)	0.23 (0.99)	0.34 (1.04)				
Perceived behavioral control				−0.06 (−0.21 to 0.10)	0.54	.46	0.001
Pre	5.31 (1.21)	5.38 (1.15)	5.23 (1.26)				
Post	5.68 (1.14)	5.72 (1.10)	5.63 (1.18)				
Change	0.37 (0.81)	0.34 (0.73)	0.40 (0.89)				

a FAQ: frequently asked questions.

b The 95% CI for the differences in mean change scores between the GPT-4o and frequently asked questions versions.

c *F* test values from univariate ANOVA tests.

### Interaction Between the Information Model and Age

We further explore whether the effect of the information model on change in attitude (outcome evaluation) depended on individual differences (eg, demographics and cultural factors). Results from a series of separate linear regressions indicated that several individual factors significantly moderated the impact of the model on attitude change.

**Figure 1.**
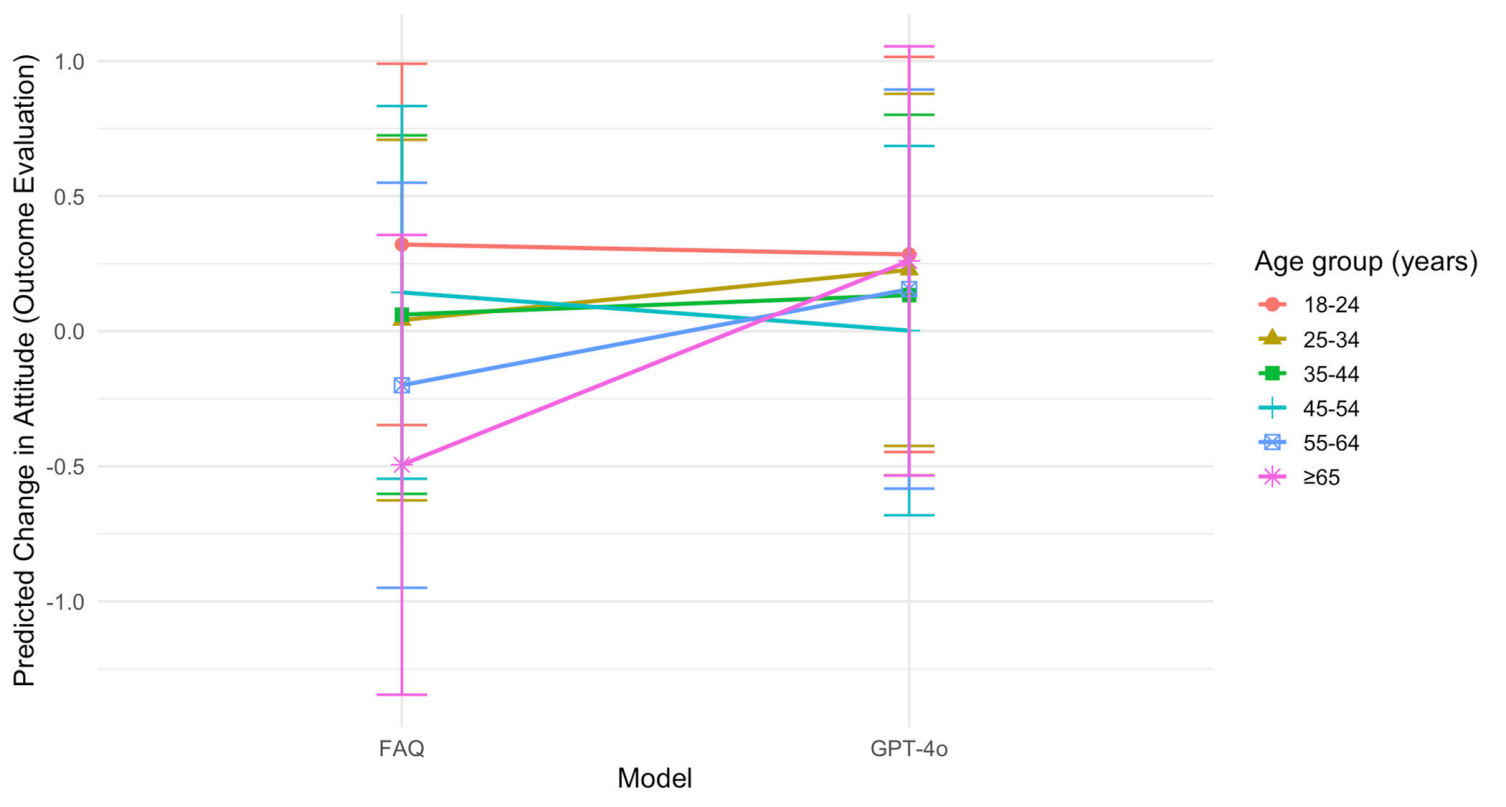
Interaction between model and age on change in attitude (outcome evaluation) toward clinical trial participation. FAQ: frequently asked questions.

A significant interaction was observed between model and age when controlling for other demographic variables (*F*_5,394_=2.25; *P*=.04). Pairwise comparisons of adjusted means showed that participants aged ≥65 years who viewed the GPT-4o content reported a significantly greater change in attitude than those who viewed the FAQ (mean difference 0.87, SE 0.33; *t*_394_=2.62; *P*=.009). A similar pattern was observed in the 55 to 64 years age group, although the difference was marginally significant (mean difference 0.50, SE 0.26, *t*_394_=1.93; *P*=.05). No other simple comparisons reached significance. When additional individual differences—including health literacy, trust in the health care system, cultural factors, perceived fears, perceived discrimination, and prior experience with clinical trials—were included as covariates, the overall interaction between model and age became nonsignificant (*F*_5,379_=1.62; *P*=.15); however, pairwise comparisons still indicated that participants aged ≥65 years who viewed the GPT-4o stimuli showed a significantly higher change in attitude than those who viewed the FAQ (mean difference 0.75, SE 0.33, *t*_379_=2.26; *P*=.02; Figure 1).

### Interaction Between the Information Model and Race

**Figure 2.**
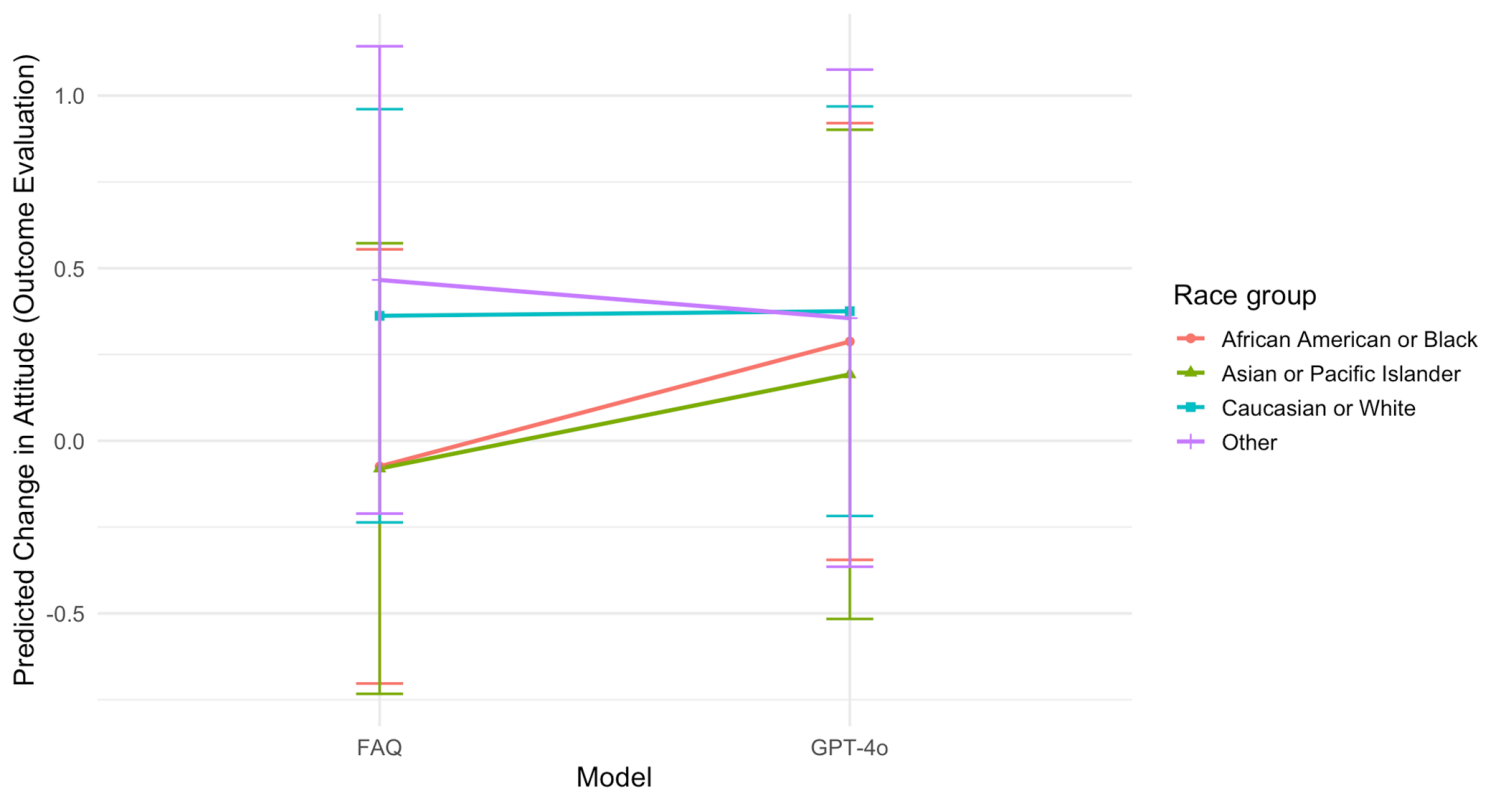
Interaction between model and race on change in attitude (outcome evaluation) toward clinical trial participation. FAQ: frequently asked questions.

The interaction between model and race was not significant overall (*F*_3,383_=1.78; *P*=.15). However, pairwise comparisons revealed that among African American and Black participants, those who viewed the GPT-4o content experienced a significantly greater change in attitude than those who viewed the FAQ (mean difference 0.36, SE 0.15; *t*_383_=2.47; *P*=.01), even after controlling for other individual differences (Figure 2). No significant differences were observed among other racial groups.

### Interaction Between the Information Model and Culture

**Figure 3.**
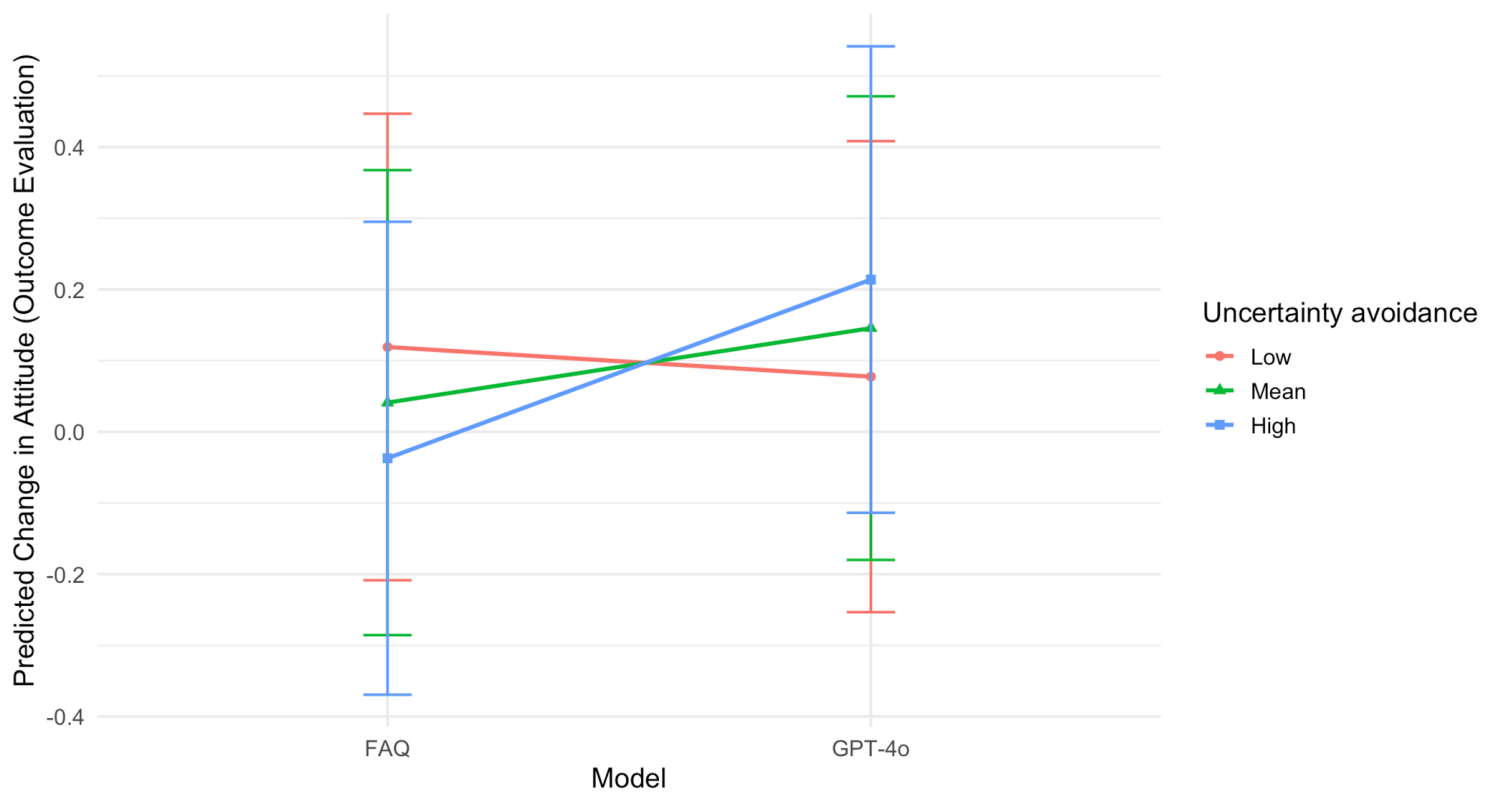
Interaction between model and uncertainty avoidance on change in attitude (outcome evaluation) toward clinical trial participation. FAQ: frequently asked questions.

A significant interaction was also observed between model and individuals’ levels of uncertainty avoidance (*F*_1,383_=4.98; *P*=.03), controlling for all other individual differences. As presented in Figure 3, participants with higher uncertainty avoidance exhibited a greater improvement in attitude toward clinical trial participation in response to GPT-4o–generated information compared with FAQ information (B=0.12; SE 0.06; *t_383_*=2.23).

### Interaction Between the Information Model and Perceived Fear

**Figure 4.**
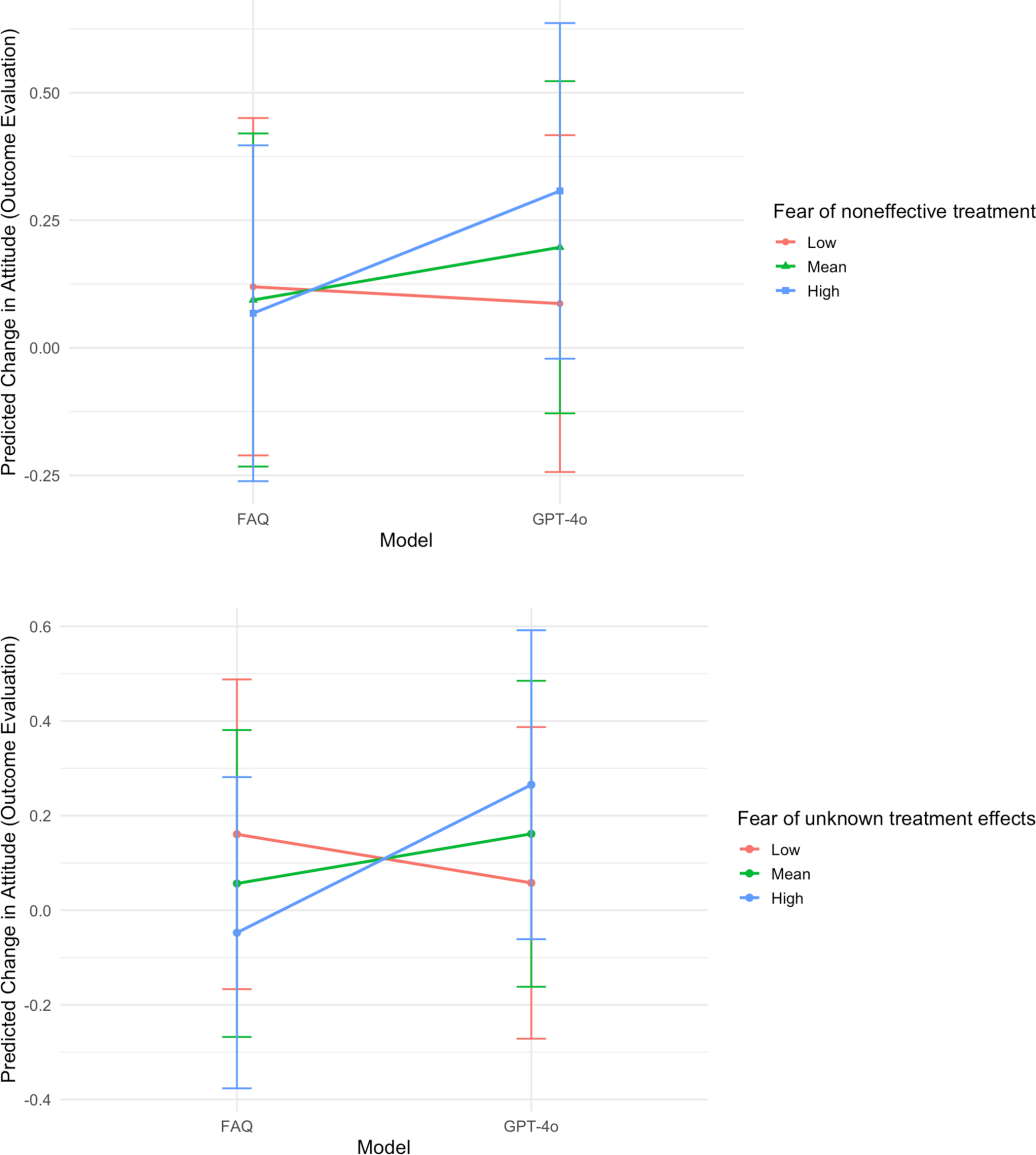
Interaction between model and perceived fears on change in attitude (outcome evaluation) toward clinical trial participation. FAQ: frequently asked questions.

Additionally, the interaction effects between the model and fear of noneffective treatment (*F*_1,383_=4.11; *P*=.04) and between the model and fear of unknown treatment effects (*F*_1,383_=9.61; *P*=.002) were significant, controlling for other individual differences. As illustrated in Figure 4, as the levels of fear of noneffective treatment (B=0.11; SE 0.05; *t_383_*=2.03) or fear of unknown treatment effects increased (B=0.21; SE 0.07; *t*_383_=3.10), GPT-4o–generated information had a progressively greater effect on improving the attitude toward clinical trial participation compared with FAQ information.

### Effects on Intention to Participate

The majority of participants (328/440, 75%) showed no change in their intention to participate in clinical trials before and after exposure to the experiment stimuli, with similar distributions observed across the GPT-4o (166/440, 75%) and FAQ versions (162/440, 74%). Fewer than 1 in 5 participants (75/440, 17%) showed a positive change in participation intention, defined as shifting from “no” or “don’t know” before exposure to “yes” afterward. When examined by the information model, 15.91% (35/440) of participants who viewed the GPT-4o content and 18% (40/440) who viewed the FAQ demonstrated a positive change (Table 4). However, results from a logistic regression analysis indicated that this difference was not statistically significant (B=−0.16; SE 0.25; Wald *χ*^2^_1_=0.4; *P*=.53; Exp(B)=0.85; 95% CI 0.52-1.40).

Informed by the TPB, a second logistic regression model was conducted by adding changes in attitudes, norms, and perceived behavioral control as predictors. Results showed that the change in attitude (outcome evaluation) toward clinical trials positively predicted a positive change in participation intention (B=0.67; SE 0.23; Wald *χ^2^*_1_=8.4; *P*=.004; Exp(B)=1.96; 95% CI 1.27-3.16). In other words, participants who exhibited a greater increase in attitude (outcome evaluation) after exposure to the experiment stimuli were more likely to demonstrate a positive change in intention to participate in clinical trials. This effect remained significant (B=1.05; SE 0.28; Wald *χ^2^*_1_=13.7; *P*<.001; Exp(B)=2.86; 95% CI 1.69-5.15) even after controlling for all individual factors. However, the information model, change in attitude (behavioral belief), changes in both types of norms, and change in perceived behavioral control were not significantly associated with positive change in participation intention (all *P* values >.05).

**Table 4.** Changes in intention to participate (N=440).

Variables and categories		GPT-4o (n=220), n (%)	FAQ^a^ (n=220), n (%)	Total, n (%)
Preintent				
Yes		126 (57)	110 (50)	236 (54)
No		21 (10)	25 (11)	46 (10)
Don’t know		73 (33)	85 (39)	158 (36)
Postintent				
Yes		153 (70)	145 (66)	298 (68)
No		27 (12)	29 (13)	56 (13)
Don’t know		40 (18)	46 (21)	86 (20)
Change				
Positive change^b^		35 (16)	40 (18)	75 (17)
Negative change^c^		15 (7)	12 (5)	27 (6)
Uncertain change^d^		4 (2)	6 (3)	10 (2)
No change^e^		166 (75)	162 (74)	328 (75)

a FAQ: frequently asked questions.

b Positive change indicates selecting “no” or “don’t know” for preintention and “yes” for postintention.

c Negative change indicates selecting “yes” or “don’t know” for preintention and “no” for postintention.

d Uncertain change indicates selecting “yes” or “no” for preintention and “don’t know” for postintention.

e No change indicates identical responses for preintention and postintention.

The regression models suggested the possibility of a moderated mediation pathway in which the information model influenced change in attitude (outcome evaluation) depending on certain individual factors (eg, age, race, uncertainty avoidance, and perceived fear), which in turn affected positive intention change. Accordingly, we conducted a series of moderated mediation analyses, each including the information model, a moderator (individual factors entered one at a time), and their interaction as predictors, change in attitude as the potential mediator, and positive intention change as the final outcome. These models controlled for other individual factors that showed significant effects in the earlier regression analyses and were used to assess whether the conditional indirect pathways were significant. The pathway analyses supported the findings from the regression models and revealed significant moderated mediation effects (Table 5). Specifically, while the information model (GPT-4o vs FAQ) did not have a direct effect on positive intention change, the GPT-4o model promoted greater attitude change among individuals with certain characteristics. These included adults aged ≥65 years, African American or Black participants, and those with higher uncertainty avoidance. In addition, individuals who reported stronger fears of noneffective or unknown treatments also showed greater attitude change. In turn, this increased attitude change enhanced positive intention change (all relevant *P* values <.05).

**Table 5.** Moderated mediation analyses.^a^

Moderator and conditional indirect effect		B^b^	SE	95% CI	*P* value
Age (years)					
Model → attitude change → positive intention change (age=>65 years)		0.53	0.20	0.14 to 0.91	.007
Race					
Model → attitude change → positive intention change (race=African American or Black)		0.22	0.11	0.01 to 0.43	.04
Uncertainty avoidance					
Model → attitude change → positive intention change (uncertainty avoidance=low^c^)		–0.03	0.06	–0.14 to 0.09	.64
Model → attitude change → positive intention change (uncertainty avoidance=mean^c^)		0.06	0.04	–0.02 to 0.15	.15
Model → attitude change → positive intention change (uncertainty avoidance=high^c^)		0.15	0.06	0.02 to 0.27	.02
Fear of noneffective treatment					
Model → attitude change → positive intention change (fear of noneffective treatment=low^d^)		–0.02	0.07	–0.16 to 0.12	.78
Model → attitude change → positive intention change (fear of noneffective treatment=mean^d^)		0.06	0.04	–0.02 to 0.15	.15
Model → attitude change → positive intention change (fear of noneffective treatment=high^d^)		0.14	0.06	0.02 to 0.27	.02
Fear of unknown treatment effects					
Model → attitude change → positive intention change (fear of unknown treatment effects=low^e^)		–0.07	0.06	–0.20 to 0.06	.27
Model → attitude change → positive intention change (fear of unknown treatment effects=mean^e^)		0.06	0.04	–0.02 to 0.14	.13
Model → attitude change → positive intention change (fear of unknown treatment effects=high^e^)		0.19	0.06	0.07 to 0.32	.002

a All models controlled for relevant covariates, including age, race, education, uncertainty avoidance, collectivism, fear of noneffective treatment, and fear of unknown treatment effects.

b Unstandardized estimates are reported.

c The low level of uncertainty avoidance refers to the mean –1 SD, the mean level refers to the sample mean, and the high level refers to the mean +1 SD.

d The low level of fear of noneffective treatment refers to the mean –1 SD, the mean level refers to the sample mean, and the high level refers to the mean +1 SD.

e The low level of fear of unknown treatment effects refers to the mean –1 SD, the mean level refers to the sample mean, and the high level refers to the mean +1 SD.

## Discussion

Guided by the TPB, this study examined for whom and in what ways LLM-generated responses to clinical trial FAQs influence attitudes, perceptions, and intentions toward trial participation. Compared with standard FAQ answers, GPT-4o–generated responses significantly improved attitudes toward clinical trials among older adults, Black participants, those with higher risk aversion, and those expressing greater fear of unknown or ineffective treatments. These results are particularly promising, as these groups are consistently underrepresented in clinical trials [34-36], and concerns about treatment uncertainty remain a major participation barrier [15]. The findings also align with prior research emphasizing the importance of empathy [37] and socially oriented communication strategies [6,38] in engaging older adults and other hesitant populations.

This study has several limitations. First, participant distribution across age and racial groups was uneven, with small sample sizes in some categories, which may have reduced statistical power for subgroup analyses and limited the generalizability of findings to underrepresented populations. Future research will aim to recruit participants representing greater linguistic and cultural diversity. Second, most participants reported English as their primary language; future studies should include more linguistically and culturally diverse samples to assess GPT-4o’s performance across different language contexts. Additionally, this study focused solely on how information about clinical trial participation is presented—only one of many factors influencing individuals’ decisions to enroll. This focus may partly explain the lack of significant changes in participation intention. Furthermore, whether a positive intention change results in actual behavioral change, that is, from refusing to joining a clinical trial, requires further research. Nonetheless, consistent with the TPB framework, the findings suggest that fostering more positive attitudes toward clinical trials may ultimately support greater participation. Further research is needed to explore additional ways AI can contribute to improving clinical trial engagement.

## Supplementary material

10.2196/87446 Multimedia Appendix 1 Questions, frequently asked questions sources, and GPT-40–generated answers for the questions.

## Abbreviations

AI: artificial intelligence
FAQ: frequently asked questions
LLM: large language model
TPB: Theory of Planned Behavior
