# Supplementary material for: Evaluating AI-Mediated Health Communication via Large Language Model–Based Frequently Asked Questions Rewriting to Foster Clinical Trial Participation: Comparative Survey Study
Source: JMIR AI. 2026 May 12;5:e87446. doi: 10.2196/87446 (PMC13167061; doi:10.2196/87446)
Supplement: Multimedia Appendix 1 [file ai-v5-e87446-s001.docx]

## **Appendix A.**

Questions, FAQ sources, and GPT-generated answers for the questions.

| **No.** | **Questions** | **GPT-generated answers** |
| --- | --- | --- |
| Q1 (24)^a^ | What happens in a clinical trial?  <https://www.pfizerclinicaltrials.com/about/frequently-asked-questions> | “In a clinical trial, doctors test new treatments to see if they are safe and work well. Volunteers get expert care, and their health is closely watched. Trials help find better medicines for everyone. You can ask questions, leave anytime, and help make a difference in healthcare!” |
| Q2. (21) | Who pays for clinical trials?<https://www.breastcancerfoundation.org.nz/breast-cancer/clinical-trials/clinical-trials-faqs> | “Many clinical trials help with travel costs, like gas, lodging, or meals. Each trial is different, so ask the research team about support options. You shouldn’t have to worry about money to help advance medicine. Your participation matters, and there may be resources to make it easier for you!” |
| Q3. (20) | What is a clinical trial?<https://clinicaltrials.uihealthcare.org/about/frequently-asked-questions-about-clinical-trials> | “A clinical trial is a research study where doctors test new medicines or treatments to make sure they are safe and work well. Volunteers help find better care for everyone. You’ll get expert medical attention, and you can ask questions, stay informed, and leave anytime if you choose.” |
| Q4. (20) | How is my safety protected if I take part in a clinical trial?<https://med.stanford.edu/cancer/clinical-trials/faq.html> | “Clinical trials are designed to be as safe as possible. Doctors carefully check treatments and closely watch your health. Safety rules protect you, and you can ask questions or leave anytime. Many people in trials help find better medicines while getting expert care. Your health always comes first!” |
| Q5. (18) | How much will I know about the benefits?<https://ccr.cancer.gov/clinical-trials/patients/faq> | “Clinical trials give you access to new treatments, expert medical care, and close health monitoring. You help doctors find better medicines for the future. Some trials may also cover costs. You can ask questions, stay informed, and leave anytime. Your participation makes a real difference in improving healthcare!” |
| Q6. (15) | Who should consider clinical trials and why?<https://www.cmoh.org/clinical-trials/clinical-trial-faqs> | “People of all ages and backgrounds join clinical trials. Some are healthy, while others have a condition doctors are studying. Every volunteer helps researchers find better treatments for everyone. You can ask questions, learn about the process, and decide if a trial is right for you. Your participation matters!” |
| Q7. (14) | What questions should I ask about participating in a clinical trial?<https://siteman.wustl.edu/research/learn-about-clinical-trials/> | “Ask about the purpose of the trial, possible benefits and risks, how long it lasts, and what costs are covered. Find out what tests or treatments you’ll receive and if you can leave anytime. It’s important to feel informed and comfortable—doctors and researchers are there to help answer your questions!” |
| Q8. (14) | What are some of the possible risks associated with taking part in a clinical trial?<https://www.sjchs.org/cancer/therapies/research-clinical-trials/frequently-asked-questions> | “Clinical trials may have risks, like side effects or treatments that don’t work as expected. But doctors closely monitor your health to keep you safe. You can ask questions, learn about the risks, and leave the trial anytime. Your health comes first, and your participation helps improve medicine for everyone!” |
| Q9. (13) | Eligibility for clinical trials<https://www.komen.org/breast-cancer/treatment/clinical-trials/> | “Each clinical trial has different rules about who can join, based on age, health, and medical history. These rules keep participants safe and help researchers get the best results. If you're interested, ask the research team—they can check if you qualify and explain everything so you can decide what’s best for you!” |
| Q10. (13) | What if I change my mind after I join?<https://www.mskcc.org/cancer-care/clinical-trials/frequently-asked-questions> | “You can leave a clinical trial anytime if you change your mind. Your health and comfort come first. Just let the research team know—they will help with next steps and answer any questions. You are always in control of your decision, and your participation still helps improve medicine!” |
| Q11. (13) | Why should I enroll in a clinical trial?<https://health.ucdavis.edu/cancer/clinical-trials/cancer-clinical-trials-booklet.pdf> | “Enrolling in a clinical trial gives you access to new treatments and expert care while helping doctors find better medicines for everyone. Your participation can improve healthcare and even save lives. You can ask questions, stay informed, and leave anytime. Every volunteer makes a difference in advancing medical research!” |

^a^The number of occurrences of the particular type of questions in the collected FAQ dataset.
